# Ecological and Functional Stratification of the Stool Microbiome Predicts Response to Immune Checkpoint Inhibitors across Cancer Types

**DOI:** 10.34133/csbj.0065

**Published:** 2026-05-14

**Authors:** Vera A. Orletskaia, Evgenii I. Olekhnovich

**Affiliations:** ^1^ Lopukhin Federal Research and Clinical Center of Physical-Chemical Medicine of Federal Medical Biological Agency, Moscow, Russian Federation.; ^2^ Moscow Institute of Physics and Technology, Dolgoprudny, Russian Federation.

## Abstract

Despite the recognized role of the gut microbiome in modulating immune checkpoint inhibitor efficacy, the ecological principles governing this relationship remain elusive. Moving beyond cataloging specific bacteria, we investigated whether general ecosystem properties determine clinical outcome. Through genome-resolved metagenomic analysis, we constructed a comprehensive catalog from 951 stool metagenomes and subsequently analyzed a curated subset of 624 samples from 11 multicancer cohorts, with melanoma (72.7%, *n* = 456) and other cancer types collectively accounting for 27.3% (*n* = 171), including gastrointestinal, non-small-cell lung, breast, ovarian, and other types. Our catalog comprises 3,816 operational genomic units and reveals the key ecological determinants of immune checkpoint inhibitor response. Clinical benefit was associated with gut ecosystems dominated by prevalent, autochthonous taxa. Indeed, the population frequency of a taxon was a positive predictor of its favorable outcome association. Functionally, responder-associated microbes were enriched in genomic capacity for complex carbohydrate metabolism, including specialized mucin degradation and amino acid biosynthesis. In contrast, nonresponse was characterized by enrichment of low-prevalence, exogenous oral and food-derived bacteria and enriched for replication-associated pathways. Our results support an ecological interpretation of the “Anna Karenina principle” in microbiomes: response is linked to a stable, functionally coherent microbial community, whereas nonresponse represents a destabilized state with high individual variability. This reframes the search for biomarkers from individual taxa to the assessment of ecosystem stability and functional coherence, providing a foundation for microbiome-targeted strategies to improve cancer immunotherapy outcomes.

## Graphical Abstract

**Figure:**
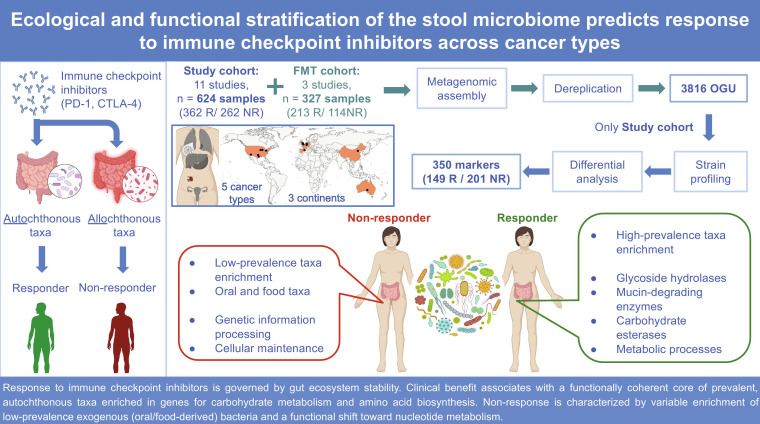

## Introduction

The introduction of immune checkpoint inhibitors (ICIs) targeting cytotoxic T-lymphocyte-associated antigen 4 (CTLA4) and programmed cell death protein 1 (PD-1) has revolutionized the treatment of advanced malignancies. However, clinical outcomes remain heterogeneous, with a significant proportion of patients exhibiting intrinsic resistance or acquiring resistance after an initial response [1]. For instance, ICIs demonstrate limited efficacy in approximately 50% of patients with metastatic melanoma [2] and are associated with a substantial risk of immune-related adverse events [3]. This underscores the critical need for predictive biomarkers to optimize patient selection and improve therapeutic outcomes.

Accumulating evidence from preclinical models [4] and clinical cohorts [5] indicates a significant association between the composition of the gut microbiome and the efficacy of cancer immunotherapy. The gut microbiome is now recognized as a pivotal modulator of systemic anti-tumor immunity. This link is further supported by interventional studies demonstrating that fecal microbiota transplantation (FMT) from responding patients can augment ICI response in germ-free mice [6] and in human patients [7]. While early research predominantly focused on melanoma [8], recent efforts are aimed at identifying conserved microbial signatures and mechanisms across diverse cancer types [9,10]. This shift is predicated on the hypothesis that fundamental host–microbiome–immune interactions are broadly relevant to immunotherapy success.

However, despite these advances, the specific biological mechanisms through which the gut microbiota influences immunotherapy response remain incompletely characterized. A recent meta-analysis consolidated evidence for stool metagenomic markers associated with positive outcomes in melanoma immunotherapy [11]. Building on this, our previous application of genome-resolved metagenomics revealed that reduced functional pathways for key microbial metabolic processes—including glycoside hydrolase-encoding genes, amino acid metabolism, and acetate synthesis via the Wood–Ljungdahl pathway—were associated with diminished efficacy of immunotherapy in metastatic melanoma [12]. Nevertheless, it remains unclear how the broader ecological principles governing the human gut ecosystem relate to immunotherapy outcomes. Furthermore, the critical question of whether the functional and ecological organizational principles represent a universal hallmark of a favorable gut ecosystem across diverse cancer types remains unanswered.

By constructing a comprehensive catalog of operational genomic units (OGUs) and analyzing a large, multicancer cohort spanning 624 metagenomic samples from 11 studies across 5 continents, with melanoma predominating (72.7%, *n* = 456) and other cancer types collectively accounting for 27.3% (*n* = 171), we established fundamental microbial determinants of immunotherapy response. Through this approach, we discovered that clinical benefit is associated with a conserved ecosystem of prevalent, autochthonous taxa exhibiting genomic capacity for complex carbohydrate metabolism and amino acid biosynthesis. In contrast, nonresponse manifests as a distinct ecological state characterized by the enrichment of low-prevalence allochthonous microbes (including oral and food-associated bacteria), by the disruption of core biosynthetic pathways, and by a shift toward capacity of nucleoside metabolism. This functional profile potentially reflects a move away from host-supportive symbiosis toward a state geared for microbial proliferation. Collectively, our findings underscore that a stable gut ecosystem structure and preserved metabolic capacity are fundamental requirements for treatment success, thereby revealing actionable targets for microbiome-modulating strategies to overcome resistance to cancer immunotherapy.

## Results

### Introduction to OGUs

Metagenomic sequencing enables the reconstruction of microbial genomes directly from complex microbial communities, yielding metagenome-assembled genomes (MAGs) that approximate the genetic repertoire of individual population members. However, due to natural strain variation and differences in assembly quality across samples, a single species may be represented by multiple highly similar MAGs, leading to redundancy that can obscure ecological and functional interpretations. To address this, we introduce the concept of OGUs—a data-driven abstraction that groups MAGs into nonredundant clusters based on a threshold of 98% average nucleotide identity (ANI). Following dereplication, each resulting cluster can be considered a representative, nonredundant MAG that serves as a reference sequence. However, to avoid terminological confusion when moving from catalog construction to downstream analyses, such as generating abundance tables, functional annotation, or ecological interpretation, we hereafter refer to these clustered units as OGUs. This framework clarifies that while the underlying genomes are derived from MAGs, the OGU abstraction is specifically designed for comparative community analysis.

This approach has a number of additional advantages. OGUs are analogous to the operational taxonomic units commonly used in amplicon-based (e.g., 16S ribosomal RNA gene) studies, which are typically defined by the sequence similarity of a single marker gene and provide only taxonomic information. In contrast, because each OGU corresponds to a genome, it enables the assignment of not only taxonomic labels but also rich functional annotations, such as metabolic pathways, carbohydrate-active enzymes, ecological traits, and genome-scale metabolic models, as well as information on possible exogenous origins. The term OGU was first introduced by Zhu et al. [13] to describe operational units obtained by mapping reads to a reference catalog. Here, we generate OGUs directly from metagenomic assembly and dereplication, an approach that more faithfully reflects the core concept of an “operational unit” built from the data themselves. Thus, an OGU serves as a multilayered data structure that integrates both biological and bioinformatic dimensions. By constructing OGUs directly from the metagenomic data at hand, rather than mapping reads to an external reference, we ensure that the units are specifically tailored to the studied cohorts, capturing population-specific genomic features. This approach transforms the OGU into a versatile functional abstraction: it is simultaneously a biologically meaningful approximation of a species, a computationally tractable entity for statistical analysis, and a scaffold for aggregating diverse annotations. Consequently, OGUs provide a seamless foundation for subsequent ecological and functional profiling, enabling a holistic investigation of microbiome–host interactions. In this study, we leverage the OGU framework to systematically characterize the stool microbiome of cancer patients and identify features associated with response to ICIs.

### Construction of an OGU catalog for cancer immunotherapy studies

**Fig. 1.**
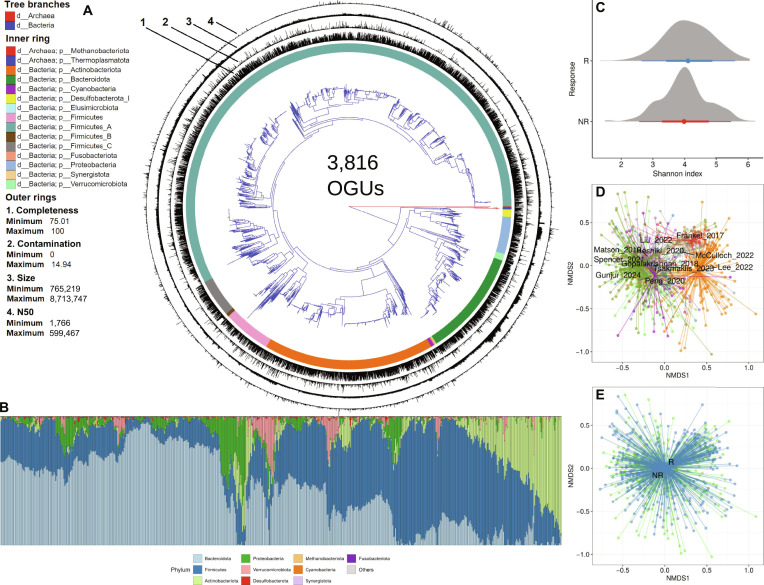
Gut microbiome catalog and associations with immunotherapy outcomes. (A) Phylogenetic diversity and quality metrics of the nonredundant metagenomic catalog. An approximate maximum likelihood phylogenetic tree was constructed from 3,816 metagenome-assembled genomes (MAGs) using a concatenated alignment of CheckM 43 universal single-copy marker proteins. The tree topology reveals evolutionary relationships across the catalog; branch colors indicate taxonomic kingdom. Concentric rings provide additional metadata: the inner ring displays phylum-level classification; outer rings visualize key genome quality metrics for each MAG, enabling direct assessment of assembly quality alongside taxonomic identity. (B) Compositional profile of the analyzed cohorts. The bar plot displays the relative abundance of bacterial phyla across the 624 baseline stool samples from 11 studies, ordered by dataset. (C) Comparison of microbial community alpha diversity via the Shannon index between responders (R) and nonresponders (NR) to immunotherapy. (D and E) Multivariate analysis of community structure via beta diversity. Nonmetric multidimensional scaling ordination based on Bray–Curtis dissimilarity reveals that samples cluster primarily by source dataset (D). Despite this dominant technical variation, a subtle but statistically significant separation by immunotherapy response is detectable (E).

To identify general ecological principles of ICI response, we constructed a comprehensive, high-quality genomic catalog of the gut microbiome from cancer patients. We integrated 951 stool metagenomes from multiple cohorts and applied quality control, retaining approximately 13 billion high-quality reads (mean ± SD: 13 ± 8 million per sample) for genome assembly. We assembled a nonredundant catalog of 13,227 MAGs. Dereplication at 98% nucleotide identity reduced these to 3,816 nonredundant OGUs—representing genome bins—with a mean completeness of 91.9% ± 6.8% and contamination of 2.13% ± 2.77% (Table S1). Adhering to Genomic Standards Consortium standards, the collection comprises 2,348 high-quality and 1,201 medium-quality OGUs, providing a foundation for taxonomic and functional profiling. Taxonomically, the catalog spans 12 phyla and is dominated by Firmicutes (2,540 genomes, 66.6%), Actinobacteria (646 genomes, 16.9%), and Bacteroidetes (402 genomes, 10.5%), with representation from Proteobacteria (140 genomes, 3.7%) and other phyla (88 genomes, 2.3%) (Fig. 1A and B). The catalog includes 11 archaeal genomes, reflecting the phylogenetic diversity of the gut ecosystem in an oncology setting.

To enable in-depth biological interpretation, each OGU was additionally annotated for functional content and potential origin. Functional annotation was performed against the Kyoto Encyclopedia of Genes and Genomes (KEGG) and Carbohydrate-Active Enzymes (CAZy) databases, providing insights into metabolic pathways and carbohydrate-active enzyme repertoires. To trace potential exogenous sources, we clustered the OGUs with reference genomes from nonintestinal body sites (oral cavity, skin, and vagina) [14] and from food metagenomes [15] at 95% ANI. This multilayered annotation transforms each OGU into a richly characterized data structure, integrating taxonomic identity, functional potential, and ecological origin.

For subsequent association analyses with clinical outcomes, we curated a cohort of 624 samples from 11 independent studies, including only patient metagenomes collected before immunotherapy initiation and excluding FMT trials to avoid confounding (Table S2). Relative abundance profiling was performed using this catalog to ensure consistent genomic resolution across all samples (Table S3). This comprehensive, publicly available catalog represents a key resource for studying host–microbiome interactions in cancer immunotherapy and enables reproducible ecological analysis across multicancer cohorts. Notably, the catalog incorporates FMT data, including samples from cancer recipients (before and after immunotherapy), from therapy-responsive donors [7,16], and from healthy donors [17]. Beyond expanding the catalog, these data provide a resource for future studies investigating the impact of FMT on immunotherapy outcomes through high-resolution strain tracking.

### Alpha and beta diversity analysis

We first assessed whether overall microbial community alpha diversity, measured by the Shannon index, differed between responders (R) and nonresponders (NR). Mean alpha diversity was comparable between groups (R, 4.13 ± 0.76; NR, 4.00 ± 0.75, mean ± SD), with no statistically significant difference detected (linear mixed-effects model with dataset as a random intercept, *P* = 0.065; Cohen’s *d* = 0.16, 95% confidence interval [CI] [0, 0.32]; Fig. 1C). As expected, dataset source accounted for substantial variance (*P* < 2.2e−16), justifying our modeling approach. The fixed effects (response and cancer type) explained only a low fraction of variance (marginal *R*^2^ = 0.02), while the full model including random effects captured moderate heterogeneity (conditional *R*^2^ = 0.33). Cancer type had no significant effect (*P* = 0.517). These results indicate that alpha diversity, as a global metric, is not a predictor of clinical outcome in our cohort, prompting us to investigate more specific compositional features.

To evaluate the association between stool microbiome composition and response to ICI therapy, we performed a multivariate analysis of community structure. We first tested for homogeneity of multivariate dispersions (PERMDISP) to assess whether the variability in community composition differed between groups. Significant differences in dispersion were observed across datasets (*F* = 4.41, *P* < 0.001) and cancer types (*F* = 14.88, *P* < 0.001), while no such difference was detected between R groups (*F* = 0.04, *P* = 0.84). These results justify the need for stratification in subsequent analyses and support the validity of within-stratum comparisons. The dataset exhibited extreme sparsity (96% zeros) and was dominated by rare taxa: half of all taxa occurred in fewer than 1.1% of samples, and only 53 taxa (1.4%) were present in more than 30% of samples. After stratifying by dataset to account for study-specific effects, we performed permutational multivariate analysis of variance (PERMANOVA) using 3 distinct distance metrics. A statistically significant association between baseline microbiome structure and treatment response was first confirmed using Bray–Curtis dissimilarity (*F* = 1.38, *R*^2^ = 0.002, *P* = 0.049). Furthermore, analyses using phylogenetically informed metrics yielded stronger associations. Weighted UniFrac showed a more pronounced effect (*F* = 2.93, *R*^2^ = 0.005, *P* = 0.015), and the strongest signal was detected using a distance derived from Phylogenetic Isometric Log-Ratio (PhILR) transformation (*F* = 4.26, *R*^2^ = 0.007, *P* = 0.014). Ordination plots based on these distances (Fig. 1D and E) aligned with the PERMANOVA results, showing primary clustering by dataset alongside a subtler, yet discernible, separation by immunotherapy response.

### Differential abundance analysis with MaAsLin2

To identify microbial taxa associated with immunotherapy response, we performed differential abundance analysis using MaAsLin2 with mixed-effects models, including dataset as a random intercept to account for interstudy heterogeneity. This analysis was conducted on datasets Frankel_2017, Gopalakrishnan_2018, Spencer_2021, McCulloch_2022, Gunjur_2024, Heshiki_2020, and Liu_2022, adjusting for key covariates (age and gender) where applicable (Fig. S1 and Table S4). The analysis revealed a core set of consistent associations, as well as several context-dependent signatures linked to specific cancer types. The most multicancer biomarker was the genus *Fusicatenibacter*, specifically the species *Fusicatenibacter saccharivorans*, which was significantly enriched in the R group across all cohorts analyzed, even after adjusting for gender and age. In contrast, NR consistently exhibited an aberrant profile characterized by enrichment of Proteobacteria—a hallmark of inflammation and ecosystem instability—and the species *Parasutterella excrementihominis*, which emerged as a multicancer marker in NR. Several other well-known beneficial taxa exhibited significant but context-dependent associations. The genus *Blautia* and its species *Blautia wexlerae* were positively associated with response specifically in the melanoma cohort. Similarly, *Faecalibacterium prausnitzii* was a significant marker in melanoma, but this association did not persist in the “other cancers” cohort after covariate adjustment. The genus *Gemmiger* presented a complex picture, with associations that reversed direction depending on cancer type and model adjustment; its species *Gemmiger qucibialis* was identified in opposing contexts—enriched in NR in adjusted melanoma, but enriched in R in adjusted other cancers.

### Microbial prevalence as a determinant of immunotherapy outcomes

Previous meta-analyses indicate that the most reliable microbial biomarkers of immunotherapy response are often highly prevalent, autochthonous taxa associated with intestinal health (e.g., *F. prausnitzii*) [11,12,18]. This recurring pattern suggests a broader hypothesis: a bacterium’s prevalence within a cohort may correlate with its association with a favorable outcome, as abundant resident microbes likely play key roles in maintaining immune homeostasis. In line with this, our top biomarker, the health-associated species *F. saccharivorans*, exhibited significantly higher-than-average prevalence (Wilcoxon rank-sum test, *P* = 3.169e−12), serving as a paradigmatic example. This observation led us to hypothesize that a bacterium’s prevalence across samples in a cohort directly correlates with its association with positive immunotherapy outcomes in melanoma. We propose that highly prevalent, autochthonous microbes are evolutionarily adapted for symbiotic coexistence and play integral roles in maintaining immune homeostasis. Their abundance is therefore associated with a stable gut environment that correlates with treatment success. Conversely, low-prevalence bacteria represent a heterogeneous group comprising either rare autochthonous ecosystem members, which are challenging to detect consistently, or allochthonous microbes potentially originating from external sources such as food or the upper gastrointestinal (GI) tract. Standard differential abundance analysis faces substantial limitations when evaluating these low-prevalence taxa. Statistical challenges including zero inflation (where bacteria are absent in most samples) and low abundance lead to unreliable regression coefficients with wide CIs and reduced statistical power. Consequently, rare taxa are typically filtered out prior to analysis, which reduces the total number of hypotheses tested and mechanically affects the stringency of multiple comparison corrections, ultimately limiting the discovery of associations with potentially informative allochthonous community members.

Initial correlation analysis of MaAsLin2 coefficients—recomputed without prevalence filtering to include all microbial taxa in the dataset—with microbial prevalence revealed a weak but statistically significant positive relationship (Spearman *ρ* = 0.14 ± 0.05, *P* < 0.001) across all analytical variants described in the previous analysis of taxonomic associations. To address the limitations of conventional approaches, we employed an alternative method based on Songbird [19], which we have successfully applied in previous studies [11,12]. This approach generates regression coefficients for each bacterium without prefiltering within each dataset independently, interpreted as log_2_ fold changes while accounting for underrepresented diversity. The Songbird-based correlation analysis demonstrated stronger and more consistent effects (Wilcoxon rank-sum test, *P* = 0.01): across all datasets, the correlation was positive in 10 of 11 datasets (Spearman *ρ* = 0.25 ± 0.09, adj. *P* < 0.001). The single exception was the Heshiki_2020 dataset (*n* = 11), which showed a significant negative weak correlation (Spearman *ρ* = −0.07, adj. *P* < 0.05). Interestingly, the correlation of Songbird coefficients with mean relative abundance also showed a similar significance pattern (Spearman *ρ* = 0.22 ± 0.12, adj. *P* < 0.001) with a statistically significant positive correlation for 10 of the 11 datasets, but for the Heshiki_2020 dataset (Spearman *ρ* = −0.07, adj. *P* = 0.05). Detailed correlation coefficients and *P* values for each individual dataset are comprehensively documented in Table S5.

The robustness of this relationship was further validated using linear regression modeling, which confirmed a significant positive association between taxon prevalence across the collected datasets and Songbird regression coefficients (estimate = 1.27e−03, *P* < 2.0e−16). Notably, this effect was independent of dataset identity, as terms for individual datasets showed no statistically significant effects after multiple-testing correction (all *P* > 0.05), indicating a universal phenomenon not driven by specific cancer types or cohort characteristics. A direct Spearman correlation between prevalence and Songbird regression coefficients also revealed a strong positive relationship (Spearman *ρ* = 0.25, *P* < 2.2e−16). In parallel, a linear model assessing the direct association between mean relative abundance and response demonstrated a highly significant positive effect (estimate = 0.30, *P* < 2.0e−16), with dataset terms again showing no significant influence (all *P* > 0.05), underscoring the robustness of abundance-based signals across cohorts.

To determine whether the prevalence–response association was merely a consequence of differences in mean relative abundance, we performed a partial correlation analysis controlling for mean relative abundance and dataset structure. After removing the variance explained by mean relative abundance and dataset, the relationship remained highly significant, although attenuated (Spearman *ρ* = 0.18, *P* = 2.2e−16). Linear modeling of the residuals confirmed this independent contribution (estimate = 1.69e−03, *P* < 2.0e−16). To further assess whether technical variability due to metagenome assembly quality could influence the observed association, we first evaluated the effect of OGU completeness in a mixed model and found it to be significant (*P* = 0.02), while OGU contamination was not significant (*P* = 0.76). We therefore repeated the partial correlation analysis including OGU completeness as an additional covariate in both residual-extraction models. After controlling for mean relative abundance, dataset structure, and OGU completeness, the prevalence–response association remained highly significant (Spearman *ρ* = 0.18, *P* < 2.2e−16). Linear modeling of the residuals confirmed this independent contribution (estimate = 1.31e−03, *P* < 2e−16), indicating that the association is not an artifact of assembly quality and that prevalence captures information beyond relative abundance, dataset origin, and technical completeness. To further exclude the possibility that the observed prevalence–response association is driven by rare, sparsely detected taxa, we performed a threshold-based sensitivity analysis using absolute prevalence cutoffs. We progressively excluded taxa with prevalence below increasing thresholds (1, 2, 3, 4, 5, 10, 20, 30, 40, and 50 samples) and recalculated the partial correlation between prevalence and Songbird coefficients, controlling for mean relative abundance, OGU completeness, and dataset structure. At all thresholds, the correlation remained positive and highly statistically significant (Spearman *ρ* ≥ 0.18, *P* ≤ 4.55e−31). Notably, when excluding taxa present in fewer than 5 samples (i.e., retaining only those with prevalence ≥5), the correlation increased from *ρ* = 0.18 to *ρ* = 0.20 (*P* = 3.71e−131). The correlation remained stable at higher cutoffs, including the most stringent threshold of ≥50 samples (*ρ* = 0.18, *P* = 4.55e−31), which corresponds to approximately 11% of the total sample size. Additionally, a permutation test shuffling response labels within datasets yielded an empirical *P* value of p_perm < 0.0001 (based on 10,000 permutations), confirming that the observed link is extremely unlikely to arise by chance.

Given the superior performance of Songbird in capturing the prevalence-association relationship, evidenced by stronger correlation coefficients and its robustness to confounding, we selected this approach for all subsequent analyses. This decision is further supported by Songbird’s inherent methodological advantages for compositional data, including its ability to model rare taxa, thereby providing a more comprehensive and biologically relevant assessment of microbial associations with immunotherapy outcomes.

### Discovery and validation of OGU markers predictive of immunotherapy response using Songbird

Songbird-based differential abundance analysis identified 350 marker OGUs that were significantly differentiated between R and NR groups. Among these, 149 features showed enrichment in R, while 201 were enriched in NR (Table S6). We found a statistically significant correlation of moderate strength (Spearman *ρ* = 0.63, *P* < 2.2e−16) between the mean Songbird regression coefficient for each marker and its prevalence in the dataset. Within this subset, prevalence was significantly higher in R (Wilcoxon rank-sum test, *P* < 2.2e−16). As expected, given the correlation between prevalence and abundance, this relationship was even stronger within this selected set of differentially abundant features.

Analysis of log-ratio values derived from metagenomic samples (Table S7) demonstrated clear separation between experimental groups, with distribution patterns visualized in Fig. S2A. Mixed-effects modeling with dataset as a random intercept revealed a significant positive association between R status and log-ratio values (*β* = 1.76, *P* < 0.001), confirming that R consistently exhibited higher log ratios. This indicates that a shift in the gut ecosystem toward a consortium of R-enriched taxa is a key determinant of treatment success. The dataset variable accounted for substantial random effects variance (*P* < 0.001), indicating important study-specific variations while maintaining the overall significant relationship. Visual inspection of the model’s residuals confirmed that their distribution was approximately normal, supporting the validity of the statistical inference. The fixed effects (response and cancer type) explained 16% of the variance (marginal *R*^2^ = 0.16), while the full model including random effects captured 21% (conditional *R*^2^ = 0.21). Notably, R status alone accounted for the majority of this explained variance (marginal *R*^2^ = 0.14 in a model without cancer type); the inclusion of cancer type increased the marginal *R*^2^ by 0.022, but this effect was not statistically significant (*P* = 0.07). Effect size analysis further substantiated the clinical relevance of this association, revealing a large practical effect (Cohen’s *d* = 0.82, 95% CI [0.65, 0.98]) that underscores the biological importance of the identified microbial signatures in distinguishing treatment response groups. The robustness of these findings was additionally supported by bootstrap validation, which yielded a 95% CI of [0.90, 2.47] for the fixed effect estimate.

We then assessed the robustness of this association by adjusting for additional demographic covariates in a subset of 352 samples from 7 datasets for which age and gender metadata were available. Mixed-effects modeling including age and gender as fixed effects confirmed that the positive association between R status and log-ratio values remained highly significant (*β* = 2.21, *P* < 0.001). Age showed a modest but significant negative effect (*β* = −0.02, *P* = 0.03), while gender (*P* = 0.81) and cancer type (*P* = 0.19) were not significant. Inclusion of these covariates increased the marginal *R*^2^ from 0.19 (response only) to 0.22 and the conditional *R*^2^ from 0.26 to 0.29, indicating that age explains a small additional portion of variance. The dataset random effect remained significant (*P* = 0.001). Bootstrap validation yielded a 95% CI of [0.61, 2.96] for the fixed effect estimate, and Cohen’s *d* was 1.0 (95% CI [0.77, 1.22]), further underscoring the large and robust effect of the log-ratio-based signature independent of demographic factors.

To additionally evaluate the generalizability and predictive power of this log-ratio biomarker, we performed a leave-one-group-out (LOGO) cross-validation. Logistic regression models were trained on all but one dataset and tested on the held-out cohort. The results demonstrate stable predictive performance across diverse studies, with a mean receiver operating characteristic area under the curve (ROC AUC) of 0.69 ± 0.12 (Fig. S2B). Detailed information about prediction metric scores is presented in Fig. S3. To benchmark the predictive capacity of the log ratio against simpler microbial metrics, we repeated the LOGO cross-validation using 2 alternative predictors: the Shannon diversity index and the Proteobacteria relative abundance. The Shannon index yielded a mean ROC AUC of 0.56 ± 0.08, which was significantly lower than that of the log ratio (Wilcoxon rank-sum test, *P* = 0.008). The Proteobacteria relative abundance achieved a mean ROC AUC of 0.63 ± 0.11, a difference that did not reach statistical significance (Wilcoxon rank-sum test, *P* = 0.19). Combining all 3 predictors into a single model resulted in a mean AUC of 0.70 ± 0.11, which was not significantly different from the log ratio alone (Wilcoxon rank-sum test, *P* = 0.77).

**Fig. 2.**
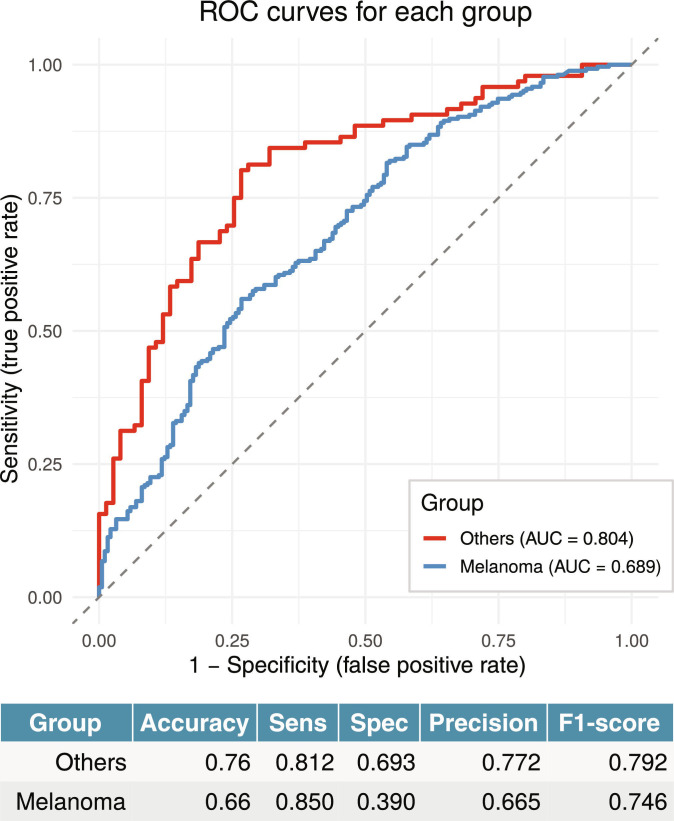
Cross-cancer validation of the operational genomic unit (OGU)-based log-ratio signature. Averaged receiver operating characteristic (ROC) curves and classification metrics for models trained on melanoma (*n* = 453) and tested on other cancer types (red) and trained on other cancers (*n* = 171) and tested on melanoma (blue). The area under the curve (AUC) values are 0.80 and 0.69, respectively; corresponding accuracy, sensitivity, specificity, precision, and F1-scores are displayed in the adjacent table.

We further assessed the transferability of the signature across cancer types by training the model exclusively on melanoma samples (*n* = 453) and testing on all other cancer types combined (*n* = 171). The model achieved an ROC AUC of 0.80. Conversely, training on the pooled nonmelanoma cohorts and testing on melanoma yielded an ROC AUC of 0.69 (Fig. 2). For comparison, the same cross-cancer evaluation was performed using the Shannon diversity index and the total relative abundance of Proteobacteria. When trained on melanoma and tested on other cancers, the Shannon index achieved an AUC of 0.59, and Proteobacteria relative abundance achieved an AUC of 0.61; in the reverse scenario (trained on nonmelanoma and tested on melanoma), the values were 0.53 and 0.57, respectively. A combined model incorporating all 3 predictors slightly improved predictive performance, yielding AUCs of 0.81 and 0.70 for the melanoma-to-others and others-to-melanoma transfers, respectively. The ROC curves for the Shannon index, Proteobacteria relative abundance, and the summary model (which combines the Shannon index, Proteobacteria relative abundance, and their log ratio) are presented in Fig. S3 for both the LOGO and cross-cancer validations.

These results confirm that the microbial log ratio captures a universal, cancer-type-agnostic signal, with particular performance when melanoma is used as the training set. While the log ratio demonstrated stable predictive performance across the cohorts analyzed in this study, its generalizability to a novel, independent dataset may vary. This potential variability arises because the log ratio is calibrated on a specific set of OGUs whose composition and representation may differ across human populations. This limited transferability precludes its immediate clinical application; accordingly, the results should be interpreted as a biological validation of the ecological signal rather than as a clinically ready predictor.

### Taxonomic and ecological profiles of the identified OGU markers

We further characterized the 350 previously identified marker OGUs through taxonomic enrichment analyses (Fig. S5). A key overarching finding was the establishment of Proteobacteria enrichment as a universal marker of nonresponse, consistently identified by both the MaAsLin2 and Songbird methods. Songbird analysis recapitulated this strong signature in the NR group (normalized enrichment score [NES] = −1.88, adj. *P* = 0.006), with core species including *Enterobacter kobei*, *Escherichia coli*, and several *Citrobacter* and *Klebsiella* species. In contrast, defining a single, universal taxonomic marker of response at the phylum level proved more complex. The most notable divergence between methods concerned the phylum Bacteroidota: it was associated with the NR group in the initial MaAsLin2 analysis but emerged as significantly enriched in the R group in the present Songbird analysis (NES = 2.24, adj. *P* < 0.001). However, the refined Songbird-based signature revealed important and consistent patterns. At finer taxonomic resolutions, the R group was enriched for the genera *Bacteroides*, *Gemmiger*, and *Faecalibacterium* (NES > 2, adj. *P* < 0.001). Notably, these R-linked markers predominantly belong to the most prevalent species in our dataset, reinforcing our earlier hypothesis that high-prevalence, autochthonous taxa are key players in a favorable outcome. Conversely, the NR group was characterized by the enrichment of *Prevotella* (NES = −1.75, adj. *P* = 0.007) and *Veillonella* (NES = −2.25, adj. *P* < 0.001). Collectively, these results consolidate Proteobacteria as a core, reproducible dysbiotic signature, alongside an increased prevalence of oral-associated taxa such as *Veillonella* and *Prevotella*—genera whose members are known to inhabit the oral cavity and may translocate to the gut environment. At the same time, our findings refine the beneficial consortium to include prevalent Bacteroidota and other high-abundance genera, a pattern that we believe is more accurately demonstrated using the Songbird framework.

**Fig. 3.**
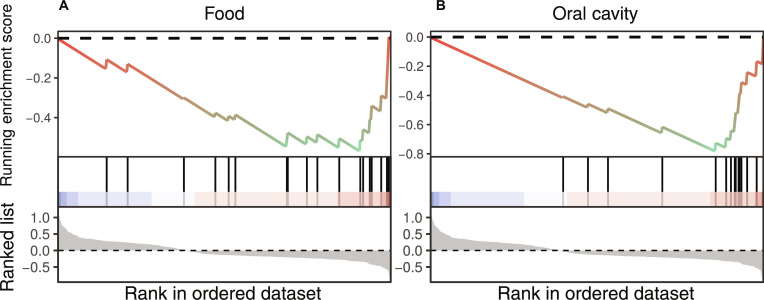
Enrichment analysis of marker operational genomic units (OGUs) across nonintestinal body sites and food origins. The enrichment analysis is presented to evaluate the distribution and enrichment of the identified marker OGUs across ranked lists of microbial taxa associated with specific bodily or dietary sources. The top panel in each subfigure displays the running enrichment score (ES) curve, where individual lines are colored according to their assigned source category. A positive ES peak indicates that the marker OGUs are significantly concentrated among taxa associated with that source and enriched in responders (R) to immunotherapy, whereas a negative score indicates concentration among taxa associated with that source and enriched in nonresponders (NR) to immunotherapy. The middle panel shows the position of the marker OGUs (vertical bars) within the ranked list of all source-associated taxa. The bottom panel illustrates the ranking metric value for each taxon, defining the order on the *x*-axis. This visualization identifies whether the marker OGUs are collectively enriched for microbial signatures typical of specific nonintestinal body habitats or dietary origins. The analysis is stratified by source type: (A) food origin and (B) oral cavity origin.

To investigate exogenous origins, we compared our 3,816 OGUs to MAGs from nonintestinal body sites and food (Fig. 3). We identified 293 OGUs clustering with nonintestinal MAGs and 91 with food-derived MAGs at the species level (95% ANI; Table S8). Strikingly, OGUs associated with NR were significantly enriched within these food-derived (NES = −2.01, *P* = 0.001) and oral-derived clusters (NES = −2.60, *P* = 6.06e−7). This pattern indicates an aberrant microbial state in NR, characterized by an increased prevalence of core enrichment we define as food-borne opportunists (e.g., *Citrobacter freundii* and *Klebsiella michiganensis*, both Proteobacteria) and oral commensals (e.g., *Veillonella parvula* and *Anaeroglobus micronuciformis*).

To quantitatively assess the overall burden of putatively exogenous bacteria, we summed the relative abundances of all OGUs that clustered with oral or food-derived MAGs across 11 datasets (*n* = 624). Mixed-effects modeling with dataset as a random intercept confirmed that NR carried a significantly higher cumulative abundance of these allochthonous taxa (*β* = −3.54, *P* = 0.0033; Cohen’s *d* = 0.24, 95% CI [0.08, 0.39]). Cancer type had no effect (*P* = 0.86). Response status alone explained 1.3% of the variance (marginal *R*^2^ = 0.013), while the full model including random effects captured 12.2% (conditional *R*^2^ = 0.12), indicating that dataset heterogeneity contributes substantially to the variability of this aggregated measure. Including cancer type as an additional fixed effect did not alter the variance explained (marginal *R*^2^ = 0.013, conditional *R*^2^ = 0.12), consistent with its nonsignificant effect. To verify that this association is independent of demographic covariates, we repeated the analysis on a subset of 352 samples from 7 datasets for which age and gender were available. After adjusting for age and gender, the significant negative association persisted (*β* = −4.06, *P* = 0.004; Cohen’s *d* = 0.33, 95% CI [0.12, 0.55]). Age (*P* = 0.26) and gender (*P* = 0.051) were not significant. Together, these results corroborate that the increased load of oral and food-associated bacteria is a consistent, albeit modest, feature of the dysbiotic state in NR, independent of age, gender, and cancer type.

### Functional profiles of the identified OGU markers

**Fig. 4.**
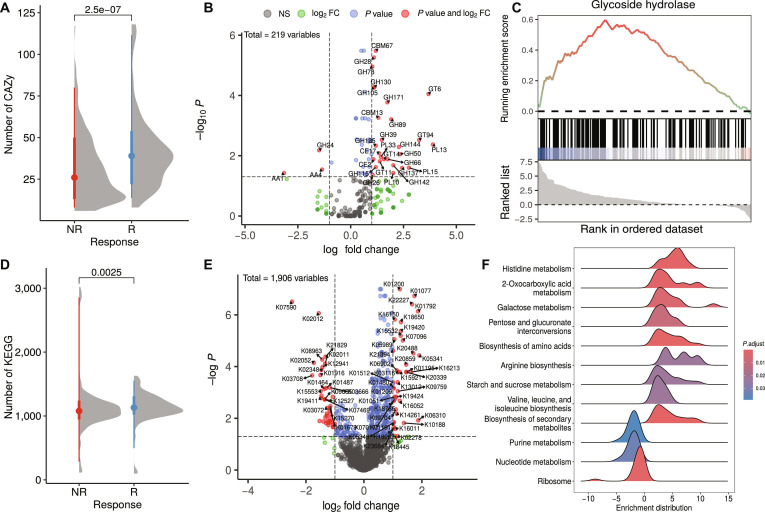
Functional profiling of immunotherapy response-associated marker operational genomic units (OGUs). Analysis of the functional potential encoded by the marker OGUs associated with responders (R) and nonresponders (NR) to immunotherapy. (A to C) Carbohydrate-Active Enzymes (CAZy) repertoire analysis. (A) The overall frequency of CAZy genes identified within the marker OGUs for each response group. (B) Specific CAZy families significantly enriched in the marker OGUs of R (positive values) and NR (negative values). (C) Enrichment analysis results highlighting glycoside hydrolase families that are coordinately enriched across the ranked list of all OGUs, with positive scores indicating enrichment in R and negative scores in NR. (D to F) Functional profile based on Kyoto Encyclopedia of Genes and Genomes (KEGG) orthology. (D) The total number of unique KEGG orthology (KO) groups present in the marker OGUs of each response group. (E) Specific KO groups showing significant differential enrichment between R and NR. (F) Pathway-level gene set enrichment analysis (GSEA) results depicting KEGG pathways significantly up-regulated (positive enrichment scores) in the R-associated marker OGUs.

Analysis of the CAZy repertoire revealed a distinct functional capacity associated with positive immunotherapy outcome. Microbial markers linked to R contained a significantly higher frequency of CAZy genes than NR-associated markers (Wilcoxon rank-sum test, *P* = 2.5e−07; Fig. 4A). Furthermore, Fisher’s exact test identified 27 CAZy families significantly enriched in the R group compared to only 3 in NR (logFC > |1|, adj. *P* < 0.05; Table S9, Fig. 4B). The enriched CAZy profile in R indicates specialized adaptation for breakdown of complex dietary and host-derived carbohydrates. This includes polysaccharide lyases PL10, PL13, PL15, and PL33, along with glycoside hydrolase families implicated in plant cell wall decomposition, such as GH26, GH28, GH78, GH105, GH115, and GH130. Notably, we observed enrichment of genes encoding GH89, GH125, and GH171—CAZy families that have been reported to participate in mucin *O*-glycan breakdown [20]. The profile also comprised several glycosyltransferases (GT6, GT11, GT14, and GT94) potentially involved in host–microbe interactions as well as specialized glycoside hydrolases for niche substrates GH39, GH50, GH66, GH137, GH142, and GH144. The saccharolytic potential was further complemented by carbohydrate esterases CE2 and CE17, alongside carbohydrate-binding modules CBM13 and CBM67.

Enrichment analysis further confirmed the overrepresentation of glycoside hydrolase families in the R group (NES = 1.33, *P* = 0.02; Fig. 4C). The core enrichment comprised 47 GH families, including hemicellulases such as GH3, GH26, GH43, GH51, and GH115; enzymes involved in pectin degradation, namely, GH28, GH78, GH88, GH105, and GH127; enzymes with putative roles in intestinal adaptation like GH2, GH20, GH29, GH89, GH95, and GH125; starch- and glycogen-active enzymes including GH13 and GH77; peptidoglycan- and chitin-active enzymes such as GH20, GH23, GH25, and GH66; and a broad array of rare and auxiliary glycoside hydrolases, for instance, GH9, GH32, GH39, GH42, GH112, and GH171 (Table S10). This diverse set underscores the multifaceted carbohydrate-degrading capacity of the R group. Collectively, these results indicate that the genomic repertoire of R-associated microbes is enriched for functions involved in carbohydrate processing. This is consistent with a potential for metabolic flexibility, including the utilization of dietary plant fibers and host-derived mucins. Similarly, the enrichment of functions targeting bacterial peptidoglycan, fungal chitin, and biofilms suggests a genomic potential that could contribute to competitive fitness within the gut ecosystem. However, direct evidence of such activities in vivo would require functional validation.

Functional annotation based on KEGG orthology revealed a distinct functional potential between the R and NR groups. A significantly higher overall number of KEGG orthology groups was observed in the R group compared to that in the NR group (Wilcoxon rank-sum test, *P* value = 0.0025; Fig. 4D). Differential abundance analysis using a Fisher’s exact test identified 43 significantly enriched genes in the R group and 42 in the NR group (logFC > |1|, adj. *P* < 0.05; Table S11, Fig. 4E). Subsequent gene set enrichment analysis (GSEA; NES > |1|, adj. *P* < 0.05; Table S12, Fig. 4F) further delineated this functional divergence. The R group exhibited a pronounced up-regulation of functional pathways central to metabolic processing, specifically encompassing ko00340 (histidine metabolism), ko01210 (2-oxocarboxylic acid metabolism), ko00052 (galactose metabolism), ko00040 (pentose and glucuronate interconversions), ko01230 (biosynthesis of amino acids), ko00220 (arginine biosynthesis), ko00500 (starch and sucrose metabolism), ko00290 (valine, leucine, and isoleucine biosynthesis), and ko01110 (biosynthesis of secondary metabolites). In contrast, the NR group was characterized by a significant enrichment of pathways fundamental to genetic information processing and cellular maintenance, namely, ko00230 (purine metabolism), ko01232 (nucleotide metabolism), and ko03010 (ribosome). This functional signature suggests that the R group microbiota is primed for active degradation of complex dietary substrates and biosynthesis of essential metabolites, whereas the NR community exhibits a profile more focused on core cellular maintenance and replication.

## Discussion

### From associations to ecological interpretation

The past decade has witnessed an exponential growth in human microbiome data, leading to the identification of numerous taxonomic associations with various pathologies. Meta-analyses consistently demonstrate reproducible, almost archetypal patterns of dysbiosis: a depletion of symbiotic microbes such as *Faecalibacterium*, *Roseburia*, and *Blautia*—highly prevalent components of a healthy microbiome—across a wide spectrum of pathologies, from inflammatory bowel diseases to metabolic syndrome and even in the context of differential responses to cancer immunotherapy [10,21–23]. Concurrently, representatives of Proteobacteria (including *E. coli*) [21,24], as well as taxa characteristic of the oral microbiota, such as *Streptococcus salivarius*, *Bifidobacterium dentium*, and *V. parvula* [25–30], have emerged as negative biomarkers in various clinical contexts. Progress in the field has not been limited to establishing correlations. The causal role of specific microbial agents, for instance, adherent-invasive *E. coli* strains in the pathogenesis of inflammatory bowel disease and colorectal cancer, has been experimentally proven in model systems [31,32].

Nevertheless, the integration of these data often fosters a simplified dualistic view: “*Faecalibacterium* is good, Proteobacteria is bad.” We posit that these classic, reproducible taxonomic markers are more a consequence than a cause, reflecting more fundamental ecological shifts within the microbial community—shifts that find their conceptual reflection in the Anna Karenina principle for microbiomes [33]. According to this principle, healthy microbial communities are characterized by a relatively uniform and stable structure (“all happy families are alike”), whereas in dysbiosis, their composition becomes highly variable and idiosyncratic (“each unhappy family is unhappy in its own way”). We suggest that this is underpinned by a universal principle governing the assembly of complex microbiological systems: the evolution of a microbial ecosystem tends toward functional competence, a goal that can be reached through multiple distinct taxonomic configurations depending on the local context [34]. This resolves any apparent tension with the Anna Karenina principle: healthy communities are indeed functionally convergent, yet this functional unity can be realized through taxonomically diverse assemblies. Thus, for each individual, it is ecologically possible to form a unique yet functionally competent community of symbionts, adapted to local conditions including diet shaped by sociocultural norms and the landscape of their immediate environment, which may explain the low taxonomic concordance when comparing cohorts from different countries and eating patterns [35].

This hypothesis for microbiomes finds direct confirmation in the context of immuno-oncology within this study. We propose an ecological interpretation of this phenomenon, based on the identified positive correlation between a taxon’s prevalence in the population and its association with a favorable response to therapy. Notably, mean relative abundance was also positively associated with response, yet prevalence captures information independent of abundance. This suggests that given 2 equally abundant taxa, the one more widespread in the population is more likely to be associated with clinical benefit. The obtained data align with the hypothesis that the “happy uniformity” of R is driven by the dominance of a highly prevalent, evolutionarily stable core of autochthonous symbionts. This core appears to be characterized by a conserved functional profile that supports the host’s metabolic needs. Patients who responded to therapy exhibit enrichment of their microbial community in genes for CAZy, particularly glycoside hydrolases known to be involved in polysaccharide metabolism, along with enrichment of KEGG pathways for amino acid biosynthesis. Together, these features point to an overall heightened metabolic potential of the R-associated microbiome. Enrichment analysis further reveals that specific CAZy families (GH89, GH125, GH129, and GH171) are significantly overrepresented in R. These families include enzymes known to participate in mucin *O*-glycan breakdown (Ram and Camille 2026). It has been hypothesized that such enzymes may facilitate a shallow, controlled degradation of the mucus layer, a trait thought to distinguish adapted commensals from aggressive colonizers [36,37]. However, whether this genomic enrichment translates into such functional activity in vivo remains to be established.

This suggests that the core is not merely increased metabolic potential but also finely tuned to its ecological niche. This hypothesis finds direct support: high intake of plant-based, fiber-rich foods is associated with improved immunotherapy outcomes and promotes the formation of a favorable microbiome in model experiments [38]. From this perspective, restoring clinical response may be linked not to specific bacterial replacement therapy, but to creating conditions that allow the reassembly of the autochthonous functional core and the restoration of cooperative interactions within the community. It is worth noting that research into FMT from responding patients to nonresponding patients substantiates this thesis [7,16]. Responding patients likely retained a larger portion of the communal microbial core, which compensated for the parts lost in NR. Furthermore, the thesis of a universally beneficial microbiome, not only in the context of immunotherapy, is supported by the effectiveness of FMT from healthy donors to improve treatment efficacy [17]. Consistent with this ecological view, factors such as an imbalanced diet, stress, and antibiotic use may disrupt community assembly processes—a disruption that, as we hypothesize, could compromise functional resilience and, consequently, diminish the capacity to support an adequate immune response [39–41]. In the newly vacated niche, competitive advantage may shift toward taxa that prioritize replication over cooperation—maximizing the use of simple resources for their own proliferation, which may be reflected in the enrichment of nucleotide metabolism pathways. In the clinical context, this ecological shift can be understood as a loss of colonization resistance, followed by the establishment of pathobionts and transient microbes, and the erosion of the immunomodulatory capacity required for an effective therapeutic response.

It is worth noting that the strength of the identified association between OGUs’ prevalence in the population and a favorable outcome is moderate. This likely reflects variability in the core microbiome (as noted above), combined with fundamental heterogeneity in the composition within the “tail” of the species abundance distribution. We hypothesize that this region harbors 2 ecologically distinct groups: true low-abundance autochthonous symbionts and transient allochthonous species originating from proximal parts of the GI tract (e.g., the oral cavity or food) or introduced with food. Confirmation of this hypothesis was obtained through an analysis based on OGUs from the assembled catalog with sets of MAGs from various human biotopes and food products. This finding is supported by our analysis, which demonstrates that taxa associated with a lack of response to therapy are enriched for OGUs that cluster together with MAGs from the oral microbiota and food. This quantitatively confirms the mixing of heterogeneous ecological signals in the low-abundance region. Nevertheless, the interpretation of these data is complicated by the fundamentally compositional nature of metagenomic data [42]. The observed enrichment of oral and food microbes in patients who did not respond to immunotherapy can be explained by 2 complementary hypotheses. First, it could be an artifact reflecting a sharp decrease in the absolute biomass of the autochthonous core. In this case, the relative increase in the proportion of oral taxa is not a consequence of their true expansion but a result of compositional distortion against the background of the “disappearance” of dominant symbionts, consistent with data on the increase in their relative abundance when overall stool microbial load decreases [43]. Second, it may reflect a true ecological colonization process. Reduced butyrate production, which often accompanies decreased diversity and the loss of key symbionts, may impair colonocyte nutrition and contribute to thinning of the mucin layer. Such changes could weaken colonization resistance, potentially allowing commensals or opportunistic pathogens from other niches to adhere and persist. This, in turn, may promote a pro-inflammatory microenvironment and systemic immune dysregulation.

### Theoretical synthesis

This work draws on the fundamental understanding that all ecosystems can be conceptualized as dissipative structures in the Ilya Prigogine sense. We propose that the gut microbiome can be understood as such an ordered system, maintained far from thermodynamic equilibrium through continuous energy flow and metabolic exchange. From an ecological perspective, this dissipative structure appears to organize when a constant flow of energy, such as carbon sources (e.g., complex carbohydrates), provides the energy substrate for microbial community growth. Within this framework, R’s ecosystem can be understood as exemplifying the principle: its stability emerges not from static taxonomy but from the continuous metabolic activity driven by energy flow—evidenced by enrichment of carbohydrate-active enzymes and amino acid biosynthesis pathways that underpin active energy dissipation through cooperative breakdown of dietary fibers and mucins. The moderate positive correlation between OGU prevalence and favorable outcome suggests that evolution has converged upon conserved ecological functions while retaining the degrees of freedom to realize them through a variety of configurations. Conversely, negative outcomes of immunotherapy could be interpreted as the ecological collapse of this dissipative structure when the energy stream is disrupted. We hypothesize that such collapse is reflected in the enrichment of allochthonous oral and food-borne microbes, which may serve as a taxonomic signature of a system that can no longer maintain its native structure. One possible interpretation is that, unburdened by cooperation, these external invaders shift toward a replication-focused strategy. The structural variability among NR thus looks like not noise but the expected behavior of a complex ecological system that has crossed a critical threshold. The Anna Karenina principle thus finds its ecological foundation. The therapeutic implications are profound: in all likelihood, adding isolated species cannot restore architecture lost. Success requires creating conditions for community reassembly and resilience preservation—whether through restoring the energy flow that sustains microbial networks or by transferring a complete community. Thus, this work provides a unifying perspective, suggesting that the microbiome supports immunotherapy not solely through its constituent parts but through its capacity to maintain itself as a coherent, dissipative whole via continuous substrate flux.

### Study limitations

While this study, being primarily theoretical in nature, provides a comprehensive ecological framework for understanding microbiome-mediated response to immunotherapy within the field of host-associated microbial ecology, several limitations should be considered when interpreting the results. While the multicancer composition of our cohort is a strength, the analysis is heavily weighted toward melanoma, which constitutes over 70% of the samples. Cross-validation strategies (training on melanoma and testing on other cancer types and vice versa) support the existence of a universal signal independent of cancer type. However, while core ecological principles are assumed to be conserved, the specific taxonomic and functional configurations of the microbiota may differ between cancer types. Future studies with larger, balanced cohorts of nonmelanoma cancers are required to validate the generalizability of the identified markers and to explore potential cancer-type-specific microbial adaptations [44].

Our analysis identifies associations between microbial community features, their functional potential, and clinical outcomes. However, the observational design of the included cohorts precludes definitive causal conclusions. Although we integrate evidence from interventional studies and mechanistic experiments from the literature to support a plausible causal pathway, future randomized controlled trials are needed to confirm that modulating the identified ecological state directly enhances treatment efficacy.

Like all metagenomic studies, our analysis is based on relative abundance data, which are inherently compositional. This limits direct inference about absolute changes in microbial biomass. The observed shifts, such as the relative increase in oral taxa in NR, could stem from a true expansion of these groups, a severe decrease in the autochthonous core biomass, or a combination of both. While we reference studies linking low microbial load to dysbiosis [43], concurrent measurements of absolute bacterial load would be required to disentangle these effects fully.

The functional profiling is based on genomic potential inferred from MAGs and gene catalogs. This reflects the community’s functional capacity but not its actual activity or metabolite production in vivo. Transcriptomic, proteomic, or metabolomic analyses of patient samples are necessary to validate that the predicted metabolic pathways are actively contributing to the host–microbe dialogue in R.

Identification of exogenous taxa relies on reference-based clustering. The identification of oral- and food-derived taxa in our catalog relies on clustering our OGU with MAGs from external reference databases at the species level (95% ANI). While this provides strong evidence for exogenous origins, it remains an approximation. Strain-level differences and ecological adaptations that occur after a microbe enters a new niche (e.g., the gut) may not be captured. Future studies employing concurrent multi-site sampling (e.g., oral and gut microbiota from the same individuals) and higher-resolution strain-tracking methods are needed to precisely map the routes of colonization, quantify the actual establishment of exogenous taxa, and understand their functional evolution in the new environment.

Due to the reliance on publicly available datasets with incomplete cohort metadata, the present study was unable to investigate the potential influence of important variables that may affect gut microbiome composition. A more comprehensive analysis of factors influencing microbiota composition is required. Beyond a meticulous characterization of basic demographic parameters (such as age and sex), future studies must account for a wider range of sociodemographic indicators and host-related factors. These include dietary habits and medication use, particularly antibiotics and proton pump inhibitors, all of which are known modulators of the gut ecosystem. If left unaccounted for, such variables may act as significant confounders or effect modifiers in the observed associations between microbial features and immunotherapy outcomes [45,46]. Regarding antibiotics, their well-documented negative impact on immunotherapy efficacy is consistent with our ecological framework. We hypothesize that this is mediated by disruption of the core microbial community, a pattern that aligns with rather than contradicts our findings. The influence of proton pump inhibitors in the specific context of immunotherapy is less clear, as these medications are not part of standard oncology treatment protocols and their use is unlikely to be systematic across cancer patients. While the importance of these factors is recognized, their systematic collection was not available across the included studies. Future prospective studies with comprehensive metadata acquisition will be essential to disentangle the effects of these variables from the ecological signals described herein and to determine whether they modulate or interact with the core principles associated with treatment response. Importantly, to enable meta-analyses in the future, patients should be stratified according to standardized Response Evaluation Criteria in Solid Tumors (RECIST) 1.1 criteria, and complete clinical metadata must be made publicly available alongside sequencing data.

## Materials and Methods

### Collection data

To create a comprehensive data resource for characterizing the gut microbial diversity in melanoma patients undergoing immunotherapy, we reconstructed a catalog of MAGs from 951 stool metagenomes across 14 public studies. This collection includes 10 melanoma-specific datasets alongside 4 datasets from other cancer types. Additionally, it incorporates metagenomic data from studies investigating FMT in melanoma immunotherapy. To ensure comprehensive coverage, we incorporated all publicly available datasets we were able to locate as of mid-2024 that contained open metadata to enable unambiguous linkage to samples deposited in public repositories. The complete dataset summary is presented in Table 1.

**Table 1.** Summary table of the characteristics of the used data

Dataset	Immunotherapy type	Cancer type	FMT	Number of samples	Sequencing platform (sequence length, bp)	Total number of reads
Frankel_2017 [47]	Anti-PD-1/anti-CTLA4	Melanoma	False	39	Illumina HiSeq 2000 (100)	1,688,904,099
Gopalakrishnan_2018 [6]	Anti-PD-1	Melanoma	False	22	Illumina HiSeq 2000 (100)	398,245,066
Matson_2018 [48]	Anti-PD-1	Melanoma	False	38	Illumina NextSeq 500 (150)	1,522,421,744
Baruch_2021 [7]	Anti-PD-1	Melanoma	True	42	Illumina HiSeq X Ten (150)	737,390,881
Davar_2021 [16]	Anti-PD-1	Melanoma	True	214	Illumina NovaSeq 6000 (150)	2,422,776,370
Spencer_2021 [38]	Anti-PD-1	Melanoma	False	134	Illumina HiSeq 2000/X (100–150)	2,028,356,126
Lee_2022 [49]	Anti-PD-1/anti-CTLA4	Melanoma	False	164	Illumina NovaSeq 6000 (150)	4,219,730,439
McCulloch_2022 [50]	Anti-PD-1	Melanoma	False	27	Illumina NovaSeq 6000 (150)	922,889,774
Tsakmaklis_2023 [8]	Anti-PD-1/anti-CTLA4	Melanoma	False	29	Illumina NovaSeq 6000 (150)	794,124,064
Routy_2023 [17]	Anti-PD-1	Melanoma	True	71	Illumina NovaSeq 6000 (150)	1,565,577,436
Peng_2020 [51]	Anti-PD-1	Cancer of part of the gastrointestinal tract (GIT)	False	40	Illumina NovaSeq 6000 (150)	1,015,338,636
Liu_2022 [53]	Anti-PD-1	Non-small-cell lung cancer (NSCLC)	False	14	Illumina HiSeq 4000 (150)	409,403,683
Heshiki_2020 [52]	Anti-PD-1	NSCLC, cancer of part of the GIT, breast cancer, ovarian cancer	False	11	Illumina HiSeq 1500 (100)	391,957,976
Gunjur_2024 [9]	Anti-PD-1/anti-CTLA4	Cancer of part of the GIT, other rare cancers	False	106	Illumina NovaSeq 6000 (150)	2,227,048,564

FMT, fecal microbiota transplantation; CTLA4, cytotoxic T-lymphocyte-associated antigen 4; PD-1, programmed cell death protein 1

The further metagenomic analysis incorporated 624 stool metagenomes obtained from 11 collected datasets (Table 1, FMT true) excluding fecal transplant data (Table 1, FMT false). Patients were stratified by immunotherapy response into 2 groups: responders (R group, *n* = 362; 58.2%) and nonresponders (NR group, *n* = 262; 41.8%). Response assessment followed RECIST 1.1, with the R group including patients showing complete response, partial response, or stable disease at least 3-month follow-up, while the NR group comprised exclusively progressive disease cases. We used the response classifications from each original study, which varied in their criteria for stable disease, and included dataset as a random intercept in all models to account for this heterogeneity. The study cohort received various immunotherapy regimens, including anti-PD-1, anti-CTLA4, or combination therapies. Patients in the Heshiki___2020 cohort received combination regimens: chemotherapy/anti-PD-1 or targeted therapy/anti-PD-1.

**Fig. 5.**
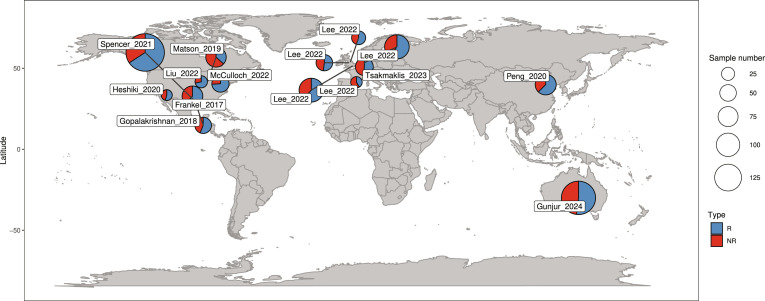
Global distribution of collected samples (*n* = 624) across countries used for metagenomic analysis. Circle size represents sample count per region, while color indicates the proportion of patients R, responsive (or NR, nonresponsive), to immunotherapy.

Cancer type distribution revealed melanoma predominance (*n* = 456; 72.7%) [6,8,38,47–50], followed by GI cancers (*n* = 82; 13.1%) [9,51,52], non-small-cell lung cancer (*n* = 15; 2.4%) [52,53], breast cancer (*n* = 4; 0.6%) [52], ovarian cancer (*n* = 2; 0.3%) [52], and other malignancies (*n* = 68; 10.8%) [9]. All samples were collected prior to treatment initiation to evaluate baseline microbiota status. The datasets are plotted on a world map as shown in Fig. 5.

### Data acquisition and preprocessing

All 951 stool metagenomic samples from 14 published studies were downloaded from the National Center for Biotechnology Information (NCBI)/European Bioinformatics Institute (EBI) databases using Kingfisher v0.4.1 [54]. All downloaded raw reads were preprocessed with fastp (v0.23.4) [55] with the following parameters: “-detect_adapter_for_pe -overrepresentation_analysis -correction -dup_calc_accuracy 6 -average_qual 30”; human DNA sequences were filtered with HISAT2 (v2.2.1) [56]. Quality control was performed using FastQC (v0.12.1) [57] and MultiQC (v1.18) [58].

### OGU catalog assembly, taxonomic profiling, and analysis

Metagenomic assembly was performed using MEGAHIT v1.2.9 [59], retaining contigs longer than 1,000 bp. Filtered contigs were aligned to metagenomic reads using HISAT2 v2.2 [56]. Binning was conducted in 2 stages: (a) initial binning with MetaBAT2 v2.12.1 [60], MaxBin2 v2.2.7 [61], and Semibin2 v2.1.0 [62], followed by (b) refinement with DAS Tool v1.1.7 to improve bin quality [63]. Bins were quality-checked with CheckM v1.2.3 [64] and dereplicated at 98% nucleotide identity using dRep v3.4.5 [65] to generate OGUs by analogy with operational taxonomic units. Zhu et al. [13] first introduced the term OGU, but our approach offers a key advantage: OGUs are generated directly from the analyzed data through genome-resolved metagenomics, rather than by mapping reads to an external reference. This more faithfully reflects the core concept of an “operational unit” built from the data itself. To our knowledge, we first applied this approach in Zakharevich et al. [12]. The resulting OGU table thus constitutes an integrative data structure: each OGU represents a genome that can be independently annotated for taxonomy, function, and origin, thereby providing a seamless, multilayered foundation for microbiome analysis. Taxonomic annotation was performed using GTDB-Tk v2.1.1 [66] with the GTDB r207 database [67]. Additional data processing utilized Samtools v1.17 [68], BEDTools v2.31.0 [69], and BBMap v39.06 [70]. Finally, OGU abundance profiles were generated by mapping reads to the OGU catalog using HISAT2 v2.2, followed by processing with InStrain v1.9.0 [71].

### Alpha and beta diversity estimation

Differences in Shannon index between R and NR were tested using a linear mixed-effects model implemented in R with the lmerTest package v3.1.3 [72]. Treatment response and cancer type were included as fixed effects (alpha diversity ~ response + cancer type + (1 | dataset)). To account for systematic variation between source studies, the dataset was included as a random intercept. The significance of fixed effects was evaluated using Satterthwaite’s approximation for degrees of freedom. The proportion of variance attributable to dataset differences was quantified by the intraclass correlation coefficient, calculated from the random intercept variance and residual variance. Marginal and conditional *R*^2^, representing variance explained by fixed effects alone and by the full model, respectively, were obtained using the r.squaredGLMM function from the MuMIn package. Assumptions of the normality and homoscedasticity of residuals were verified graphically. The effect size for the group comparison was calculated as Cohen’s *d* using the cohens_d function from rstatix package v0.7.3.

To assess the association between gut microbiome composition and response to ICI therapy, we performed a multivariate analysis of beta diversity. Prior to formal hypothesis testing, the homogeneity of multivariate dispersions was evaluated using a permutational analysis of multivariate dispersions test with the betadisper function, based on 9,999 permutations. This test assessed the key assumption of comparable within-group variation for the factors of dataset, cancer type, and response group. The main association between community structure and clinical outcome was then tested using a stratified PERMANOVA with 999 permutations. To control for dominant interstudy heterogeneity, the PERMANOVA model was applied within strata defined by the source dataset, and the overall significance was determined by combining stratum-specific results (ogu_table ~ response, strata = dataset). The analysis employed 3 complementary distance metrics to capture different ecological dimensions. Bray–Curtis dissimilarity was used as an abundance-based metric. Weighted UniFrac distance was calculated using the phyloseq package v1.52.0 [73]. Additionally, a phylogenetically informed Euclidean distance was generated by first applying the PhILR [74] transform to address data compositionality and create phylogenetically aware balances using philr package v1.34.0. For visualization, community patterns were displayed using nonmetric multidimensional scaling (NMDS) ordination based on Bray–Curtis dissimilarity. All analyses were conducted in R version v4.4.2. Multivariate procedures including betadisper, PERMANOVA, and NMDS were performed using vegan package v2.7.2 (https://github.com/vegandevs/vegan).

### MaAsLin2-differentiated taxonomic analysis framework

To identify taxa differentially associated with treatment response, we implemented a tiered analytical strategy designed to ensure robustness against dataset imbalances and confounding variables. Our cohort was characterized by a substantial imbalance between melanoma (*n* = 453) and other cancer types (*n* = 171), necessitating separate validation of markers within each subgroup. Our primary analysis employed MaAsLin2 v1.22.0 [75] within a mixed-effects model framework, with immunotherapy response or cancer type in combined analysis as a fixed effect and dataset included as a random effect. We conducted parallel analyses across 3 data partitions: the full cohort (*n* = 624), melanoma-only subset (*n* = 453), and nonmelanoma cancers (*n* = 171). To control for potential demographic confounders, we performed a secondary analysis on a restricted sample with available metadata (*n* = 353, included 7 datasets—Frankel_2017, Gopalakrishnan_2018, Spencer_2021, McCulloch_2022, Gunjur_2024, Heshiki_2020, and Liu_2022), incorporating gender and age as additional fixed effects while maintaining the same random effects structure. To overcome the limited statistical power of individual taxon analyses in sparse microbiome data, we employed a systems biology approach. We aggregated MaAsLin2 results using GSEA across taxonomic hierarchies with clusterProfiler package v4.16.0 [76], ranking features by their MaAsLin2 regression coefficients with threshold false discovery rate < 0.1. This approach amplifies coherent biological signals by accumulating weak but consistent effects across related taxa, thereby enhancing the detection of biologically meaningful patterns that might be lost in conventional single-taxon analyses. To comprehensively assess the relationship between microbial prevalence and treatment association, we conducted a correlation analysis between OGU prevalence and MaAsLin2-derived coefficients. For this specific analysis, MaAsLin2 was run without prevalence or abundance filtering (min_abundance = 0, min_prevalence = 0) to include the complete taxonomic spectrum. The association was quantified using Spearman’s rank correlation, with statistical significance determined using the cor.test function in R, followed by Benjamini–Hochberg multiple-testing correction via the p.adj function.

### Determination of OGUs associated with immunotherapy outcomes using Songbird

In this study, we intentionally deviated from conventional hypothesis-driven frameworks targeting individual microbial taxa to circumvent limitations intrinsic to metagenomic data analysis. Instead, we implemented a differential ranking approach to prioritize features based on their association strength with clinical response to immunotherapy, coupled with log-ratio transformations to quantify ecosystem-level restructuring of the gut microbiota [19]. This methodological decision was driven by the compositional nature of microbiome data, where log ratios mitigate feature interdependencies while preserving ecological interpretability, and their inherent robustness to technical biases such as uneven gram-negative/positive bias during DNA extraction or sequencing depth. This framework aligns with contemporary paradigms in microbiome research, where holistic community dynamics supersede isolated taxonomic signatures [42].

The identification of OGUs associated with immunotherapy outcomes followed an established analytical framework from our previous works [11,12]. Differential rankings were first performed using Songbird v1.0.3 [19] to identify OGUs showing relative abundance variations between experimental groups, applying a conservative absolute differential value threshold of > 0.3. For candidate OGUs that met this criterion, we subsequently calculated log-ratio abundances according to the principle described in the works of Morton et al. [19] and Fedarko et al. [77]. The statistical significance of the division of patients by response based on log ratios was determined through Wilcoxon rank-sum tests with alpha threshold = 0.05 implemented in R statistical environment v4.4.2. The biomarker selection process incorporated stringent cross-validation criteria to ensure the identification of microbial signatures. OGUs demonstrating consistent positive associations with therapeutic response across multiple datasets were retained as potential beneficial biomarkers, while any evidence of negative association with treatment outcome in any dataset resulted in automatic exclusion regardless of other positive associations. Consequently, any markers that were characteristic only of melanoma datasets were removed from the final set. This approach enabled simultaneous identification of 2 clinically meaningful biomarker categories: microbial taxa positively correlated with successful immunotherapy outcomes and those associated with adverse therapeutic responses. The methodology emphasizes reproducibility through multidataset validation and maintains rigorous standards for biomarker qualification by requiring consistent directional effects across independent cohorts.

Correlation analysis between regression coefficients and OGU prevalence was performed similarly to the analysis with MaAsLin2 regression coefficients. For this purpose, Songbird-derived coefficients for each OGU were combined with 2 global metrics: prevalence, defined as the number of datasets in which the OGU was detected, and mean relative abundance. To assess the independent contribution of prevalence while properly accounting for interstudy heterogeneity, a linear mixed model was fitted using the lmer function from the lmerTest v3.1.3 package, with the Songbird coefficient as the dependent variable, prevalence and mean relative abundance as fixed effects, and dataset included as a random intercept to absorb variation due to different cohort origins. The significance of fixed effects was evaluated using Satterthwaite’s approximation for degrees of freedom. To confirm that the observed effect of prevalence was not driven by any particular study, we tested whether adding dataset-specific fixed effects improved the model; none remained significant after multiple-testing correction, indicating a universal phenomenon independent of dataset identity.

Additional validation of the relationship was performed through 3 complementary approaches. First, a partial correlation analysis was conducted to isolate the unique contribution of prevalence after removing the variance explained by mean relative abundance and dataset structure. Residuals were extracted from 2 models: one regressing the Songbird coefficient on mean relative abundance with dataset treated as a fixed factor to absorb all between-study variation and another regressing prevalence on mean relative abundance with dataset as a random intercept. The Spearman correlation and linear modeling between these 2 sets of residuals provided an estimate of the prevalence–response association independent of both abundance and study origin. To further control for potential technical confounding due to metagenome assembly quality, the same procedure was repeated with OGU completeness included as an additional covariate in both residual-extraction models. Second, a threshold-based sensitivity analysis was performed to directly assess whether the observed association might be driven by rare, sparsely detected taxa. Using absolute prevalence cutoffs, we progressively excluded taxa with prevalence below increasing thresholds (1, 2, 3, 4, 5, 10, 20, 30, 40, and 50 samples). For each threshold, we repeated the partial correlation analysis described above, controlling for mean relative abundance, OGU completeness, and dataset structure. The correlation coefficient and its significance were recorded to evaluate the stability of the effect as the rarest taxa were removed. Third, a permutation test was performed with 10,000 iterations using the permlmer function from the predictmeans v1.1.1 package. This function randomly shuffles the response values within each dataset (preserving the original clustering structure) and refits the linear mixed model, recording the coefficient for prevalence. The empirical *P* value was calculated as the proportion of permutations in which the absolute coefficient equaled or exceeded the absolute observed value. The ranova function from lmerTest v3.1.3 was used to test the significance of the random effect (dataset) by likelihood ratio comparison.

### Validation of OGU as a candidate marker for stratifying patients by response to immunotherapy

Determined R/NR biomarkers were used to calculate log ratios, followed by statistical assessment using the lmer function from lmerTest package v3.1.3 [72] in the R statistical environment (log_ratio ~ response + (1 | dataset)). To evaluate whether the association between OGU prevalence and response-related coefficients was significant, we performed a Spearman correlation test between the mean Songbird regression coefficient for each marker and its prevalence across samples using the cor.test function. Model residuals were inspected for normality through visual examination. Marginal and conditional *R*^2^ were calculated using the r.squaredGLMM function from the MuMIn package to estimate the variance explained by fixed effects alone and by the full model, respectively. Effect size for the response group comparison was quantified using Cohen’s *d*, calculated as the difference in mean log ratio between groups divided by the pooled standard deviation. To assess the potential influence of cancer type, we also fitted an extended model including cancer type as an additional fixed effect (log_ratio ~ response + cancer_type + (1 | dataset)); the effect of cancer type was not statistically significant (*P* = 0.07). Bootstrap validation was performed using the lmeresampler package. We applied a case bootstrap approach with 1,000 iterations, resampling both individual observations and groups, to generate 95% percentile CIs for the fixed effects estimates.

Additional testing of marker OGU included investigating the ability of calculated log ratios to separate experimental groups using logistic regression. We employed 2 complementary validation strategies. First, we performed LOGO cross-validation, training on all but one dataset and testing on the held-out cohort. Second, to assess cancer-type transferability, we trained models exclusively on melanoma samples and tested on all other cancer types combined, and vice versa. All analyses were implemented using tidymodels package v1.3.0 [78].

### OGUs’ origin prediction

The presence of non-gut microbes in the stool microbiome, such as those frequently detected in the oral cavity, has been associated with a degradation of the gut microbiota [43]. This phenomenon frequently results in adverse outcomes, including various illnesses. The identification of non-gut microbes was conducted using published microbial MAGs from body sites (oral, skin, and vagina) [14] and food [15]. The body site MAGs were retrieved from http://segatalab.cibio.unitn.it/data/Pasolli_et_al.html. A total of 9,412 genomes were selected based on specific criteria, including those from the “body site” category, such as the airways, nasal cavity, oral cavity, skin, and vagina. Genomes categorized in the “age category” as “school age” (12 to 19 years), “adult” (19 to 65 years), and “senior” (65 to 70 years) were included in the analysis. Genomes from newborns (<1 year) or children (1 to 12 years) were excluded, as the gut microbiota typically reaches an adultlike phylogenetic composition by 3 years of age [79]. The selected genomes were then clustered with our reconstructed catalog via dRep v3.4.5 [65] with 95% nucleotide identity and the -ignoreGenomeQuality parameter. Genomes that passed the similarity cutoff to body site MAGs were labeled from the same source. The food MAGs were obtained from https://zenodo.org/records/13285428, where the cFMD_mags.tar.gz archive contains the folder cFMD_mags_prok with prokaryotic genomes. These 10,112 MAGs were preclustered among themselves using dRep v3.4.5 with 95% nucleotide identity and the -ignoreGenomeQuality parameter, yielding a total of 983 genomes. The subsequent source identification process was consistent with that employed for body MAGs.

GSEA was used as a summarizing tool to study the statistical relationships of OGU marker sets with immunotherapy response. This analysis was implemented using the GSEA function from the clusterProfiler package (v4.16.0) [76] and examined relationships across taxonomy, gene sets, and other traits such as presence in food or body site. For this analysis, items for each trait were ranked based on the *P* value multiplied by the sign of the Songbird regression coefficient (R/NR + *k*), where a negative sign indicates association with the NR group and a positive sign indicates association with the R group. GSEA results were visualized using GseaVis package v0.1.1 [80].

To quantitatively evaluate the overall burden of putatively exogenous taxa, we calculated for each sample the summed relative abundance of all OGUs that clustered with food- or oral-derived reference genomes at 95% ANI. This aggregated measure was then compared between R and NR using a linear mixed-effects model implemented with the lmer function from lmerTest package v3.1.3 [72]. The model included response status as a fixed effect, with dataset as a random intercept to account for interstudy heterogeneity (cumulative_relative_abundance ~ response + (1 | dataset)). Cancer type was also tested as an additional fixed effect but did not reach significance. In a subset of samples with available demographic data (*n* = 352 from 7 datasets), age and gender were included as covariates. The significance of fixed effects was evaluated using Satterthwaite’s approximation for degrees of freedom. Marginal and conditional *R*^2^ were calculated with the r.squaredGLMM function from the MuMIn package to estimate variance explained by fixed effects alone and by the full model, respectively. Effect sizes were quantified as Cohen’s *d* using the cohens_d function from the rstatix package v0.7.3. To evaluate the contribution of dataset as a random effect, we performed a likelihood ratio test comparing the full mixed model (including dataset as a random intercept) against a null model with only fixed effects, using the ranova function from lmerTest package v3.1.3. A significant result (*P* < 0.05) indicates that accounting for interdataset heterogeneity substantially improves model fit. The proportion of total variance attributable to dataset differences was quantified by the intraclass correlation coefficient, calculated as the ratio of the random intercept variance to the sum of random intercept and residual variances.

### Functional annotation of OGU marker sets

Functional annotation of the OGU markers was performed using MetaCerberus v1.4.0 [81] against the CAZy [82] and KEGG [83] databases. To identify significant functional differences between marker sets, we employed a multitiered statistical approach beginning with Wilcoxon rank-sum tests to compare gene abundance distributions. Subsequently, Fisher’s exact tests with Benjamini–Hochberg false discovery rate correction < 0.05 were applied to identify significantly enriched functional features between experimental groups. Finally, GSEA was implemented to evaluate coordinated changes at the level of CAZy enzyme classes and KEGG pathways.

## Ethical Approval

For the analysis, sample identifiers from the Sequence Read Archive (SRA) archive and publicly accessible metadata from published studies were employed. The data used in this study were sourced from SRA/NCBI. All original studies obtained informed consent from participants for the publication of anonymized data. Our analysis did not require additional ethical approval, as only de-identified datasets were utilized from open sources.

## Data Availability

In this study, we used open-access data from the NCBI/EBI Sequence Read Archives, identified by the following BioProject accession numbers: PRJNA397906, PRJEB22893, PRJNA399742, PRJNA678737, PRJNA672867, PRJNA770295, PRJEB43119, PRJNA762360, PRJNA1011235, PRJNA928744, PRJNA615114, PRJNA866654, PRJNA494824, and PRJEB49516. The pipeline for OGU catalog assembly and the sampleid.txt file containing the SRA/NCBI accession numbers used are available at https://github.com/JeniaOle13/Cancer_MAGs. All initial MAGs sequences were deposited in NCBI under accession PRJNA1196825. The source code and Quarto report are available at https://github.com/JeniaOle13/cancer-biomarkers. A preprint of this work is available on bioRxiv (https://www.biorxiv.org/content/10.1101/2025.05.07.652660.abstract).
