# Supplementary material for: Ecological and Functional Stratification of the Stool Microbiome Predicts Response to Immune Checkpoint Inhibitors across Cancer Types
Source: Comput Struct Biotechnol J. 2026 May 14;35(1):0065. doi: 10.34133/csbj.0065 (PMC13173278; doi:10.34133/csbj.0065)
Supplement: Supplementary 1 — Tables S1 to 12 Figs. S1 to S5 [file csbj.0065.f1.zip › Figure_S4.pdf]

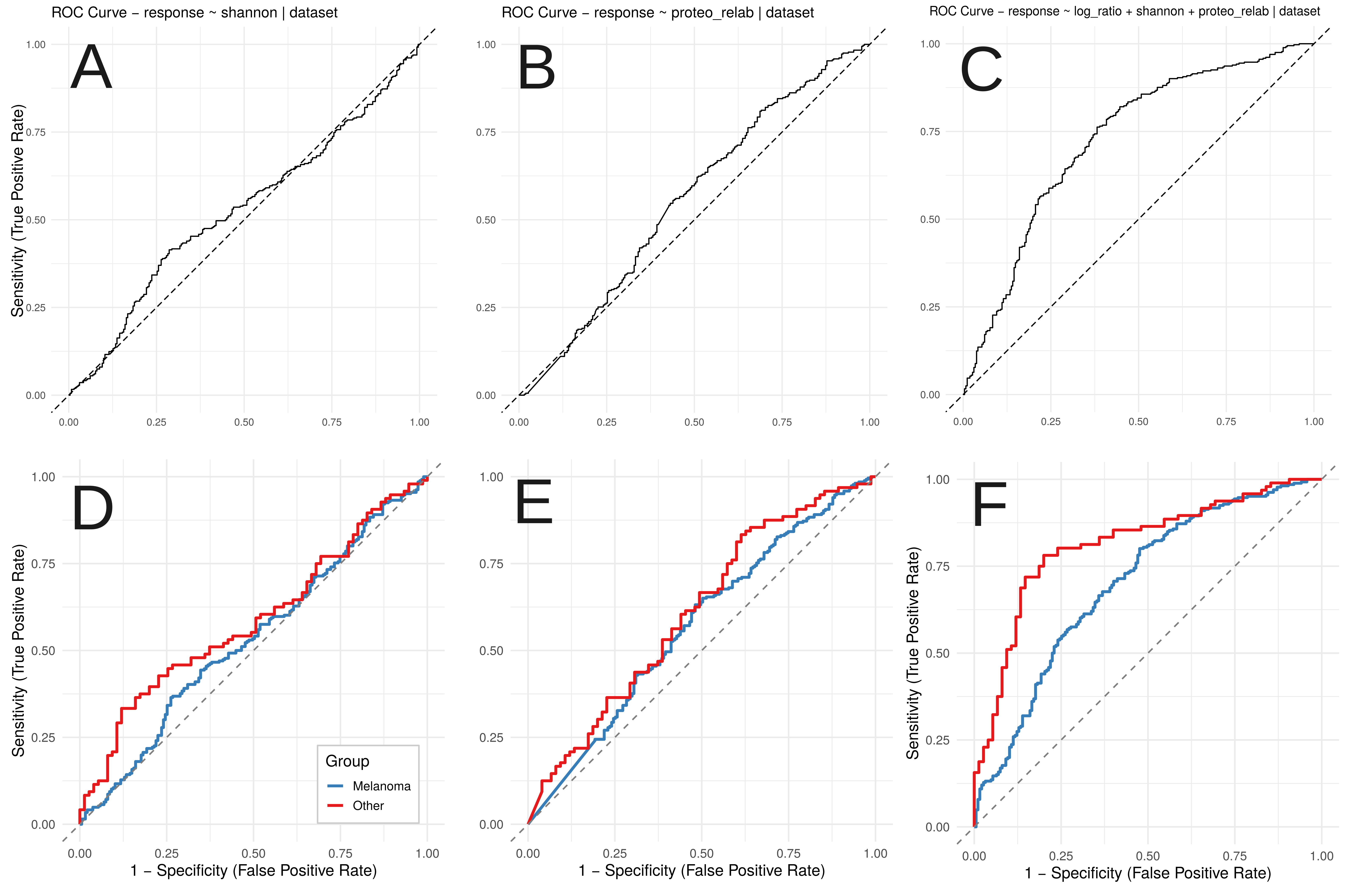

ROC Curve – response ~ shannon | dataset
ROC Curve – response ~ proteo_relab | dataset
ROC Curve – response ~ log_ratio + shannon + proteo_relab | dataset
A
B
C
D
E
F
Sensitivity (True Positive Rate)
1 – Specificity (False Positive Rate)
Group
Melanoma
Other
0.00
0.25
0.50
0.75
1.00
