# Supplementary material for: Ecological and Functional Stratification of the Stool Microbiome Predicts Response to Immune Checkpoint Inhibitors across Cancer Types
Source: Comput Struct Biotechnol J. 2026 May 14;35(1):0065. doi: 10.34133/csbj.0065 (PMC13173278; doi:10.34133/csbj.0065)
Supplement: Supplementary 1 — Tables S1 to 12 Figs. S1 to S5 [file csbj.0065.f1.zip › sm.docx]

**Supplementary information**

Table S1. Genome quality statistics for the non-redundant catalog of 3,816 MAGs, including completeness, contamination, and taxonomic classification.

Table S2. Clinical and technical metadata for the curated cohort of 624 baseline stool metagenomes from 11 studies, including patient response status, cancer type, age, gender, and dataset source.

Table S3. Normalized relative abundance matrix of 3,816 OGUs across 624 samples, generated by mapping reads to the custom MAG catalog for consistent taxonomic profiling.

Table S4. Differential abundance analysis with MaAsLin2 of microbial taxa associated with immunotherapy response.

Table S5. Dataset-specific results from the correlation analysis between microbial prevalence and Songbird-derived association coefficients with immunotherapy response.

Table S6. Complete set of 350 marker OGUs identified by Songbird differential ranking, annotated with their direction of association (R/NR) and regression coefficients.

Table S7. Sample-level log-ratio values derived from the 350 marker OGUs, serving as the core predictive feature for immunotherapy response stratification.

Table S8. Results of the exogenous origin analysis showing catalog OGUs that cluster at 95% Average Nucleotide Identity (ANI) with reference MAGs from oral, other body sites, and food sources.

Table S9. Differential enrichment of CAZy families in the genomes of R- versus NR-associated marker OGUs (Fisher’s exact test, FDR-corrected).

Table S10. GSEA results showing GH families coordinately enriched in the R-associated marker OGUs.

Table S11. Differential abundance of KO groups in the genomes of R- versus NR-associated marker OGUs (Fisher’s exact test, FDR-corrected).

Table S12. Pathway-level GSEA of KEGG metabolic pathways in the genomes of R- versus NR-associated marker OGUs.

Figure S1. Functional enrichment analysis of taxonomic shifts identified by differential abundance analysis. Results of the functional enrichment analysis for taxonomic shifts identified as significant by the MaAsLin2 differential abundance analysis. Positive associations, where a taxon is enriched in the target condition, are shown in blue. Negative associations, where a taxon is depleted, are shown in red. Panel (A) displays significant associations at the species level, Panel (B) at the genus level, and Panel (C) at the family level. Statistical significance levels are denoted as follows: ***p < 0.001; **p < 0.01; *p < 0.05; ∙ p < 0.1; ° p < 0.25.

Figure S2. Validation of the log-ratio biomarker for immunotherapy response. (A) Distribution of log-ratio values, calculated from marker OGUs, for R and NR patients. (B) LOGO cross-validation results assessing the predictive performance of the log-ratio-based biomarker. The line indicates the mean AUC across all datasets (0.69 ± 0.12).

Figure S3. Comprehensive performance metrics of the log-ratio biomarker across independent cohorts. The figure presents detailed prediction metrics from the LOGO cross-validation analysis. Metrics include the area under the receiver operating characteristic curve (AUC), accuracy, sensitivity, specificity, precision, and F1-score.

Figure S4. Averaged ROC curves evaluate the discriminatory power of the Shannon index, the relative abundance of Proteobacteria, and a summary model integrating these features with the log‑ratio based on 350 marker OGUs. Panels (A–C) show results from cross‑validation stratified by dataset. Panels (D–F) display results from cross‑validation stratified by cancer type; in these panels, red curves represent models trained on melanoma samples (n = 453) and tested on other cancer types, while blue curves represent models trained on other cancer types (n = 171) and tested on melanoma. (A, D) Predictions based on the Shannon diversity index. (B, E) Predictions based on the total relative abundance of Proteobacteria. (C, F) Predictions based on a combined model incorporating the Shannon index, Proteobacteria abundance, and the log‑ratio of the 350 marker OGUs.

Figure S5. Taxonomic enrichment analysis characterizing the 350 marker OGUs identified by Songbird. The results of a Gene Set Enrichment Analysis (GSEA) are presented to evaluate the coordinated enrichment patterns of the identified marker Operational Genomic Units (OGUs) across phylogenetically ranked lists. The top panel in each subfigure displays the running Enrichment Score (ES) curve, where individual lines are colored according to their corresponding taxonomy. The position of the maximum ES (peak) indicates where the marker set is most concentrated within the ranked list; a positive score denotes enrichment at the top of the ranking (associated with the R group), while a negative score denotes enrichment at the bottom (associated with the NR group). The middle panel shows the distribution of the marker OGUs (vertical bars) across the same ranked list of all taxa, indicating where members of the significant set appear. The bottom panel illustrates the value of the ranking metric for each taxon in the list, which forms the basis for the ranking on the x-axis. This integrated visualization identifies biologically coherent taxonomic groups whose members are non-randomly distributed towards the extremes of the phenotypic ranking. The analysis is shown at distinct taxonomic resolutions: (A) phylum level, (B) genus level, and (C) species level.
