# Supplementary figures and images for: Ecological and Functional Stratification of the Stool Microbiome Predicts Response to Immune Checkpoint Inhibitors across Cancer Types
Source: Comput Struct Biotechnol J. 2026 May 14;35(1):0065. doi: 10.34133/csbj.0065 (PMC13173278; doi:10.34133/csbj.0065)

A

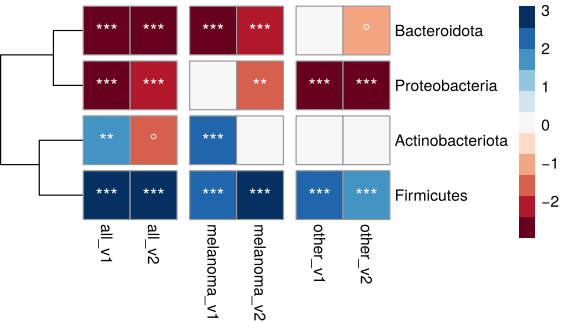

B

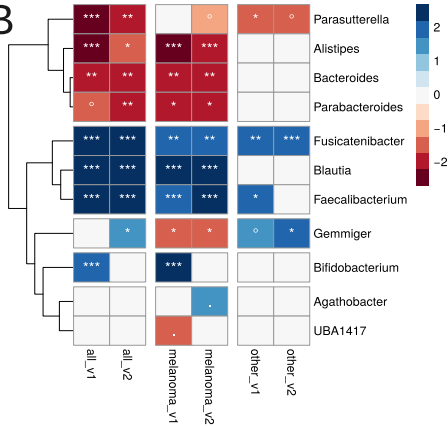

C

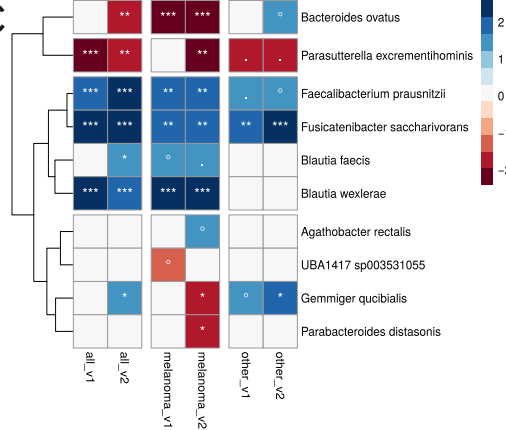

Supplement: Supplementary 1 — Tables S1 to 12 Figs. S1 to S5 [file csbj.0065.f1.zip › Figure_S1.pdf]

**A**

Response

NR

R

-5

0

5

Log ratio

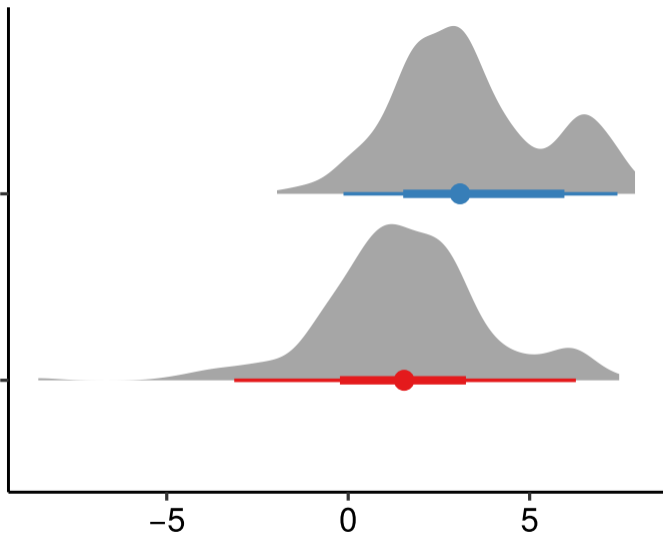**B**

sensitivity

1.00  
0.75  
0.50  
0.25  
0.00

0.00

0.25

0.50

0.75

1.00

1 - specificity

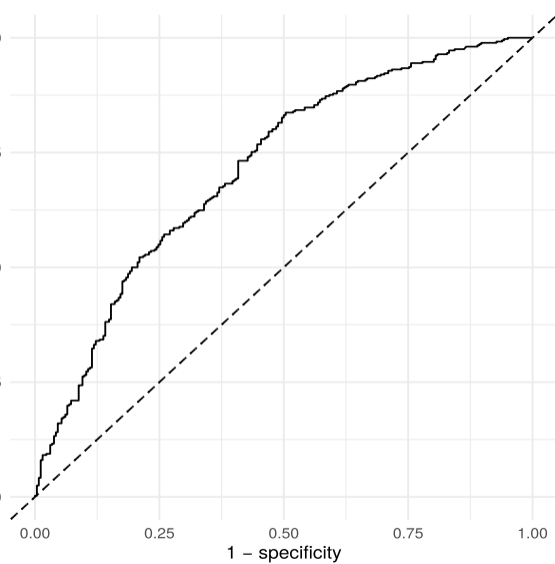

Supplement: Supplementary 1 — Tables S1 to 12 Figs. S1 to S5 [file csbj.0065.f1.zip › Figure_S2.pdf]

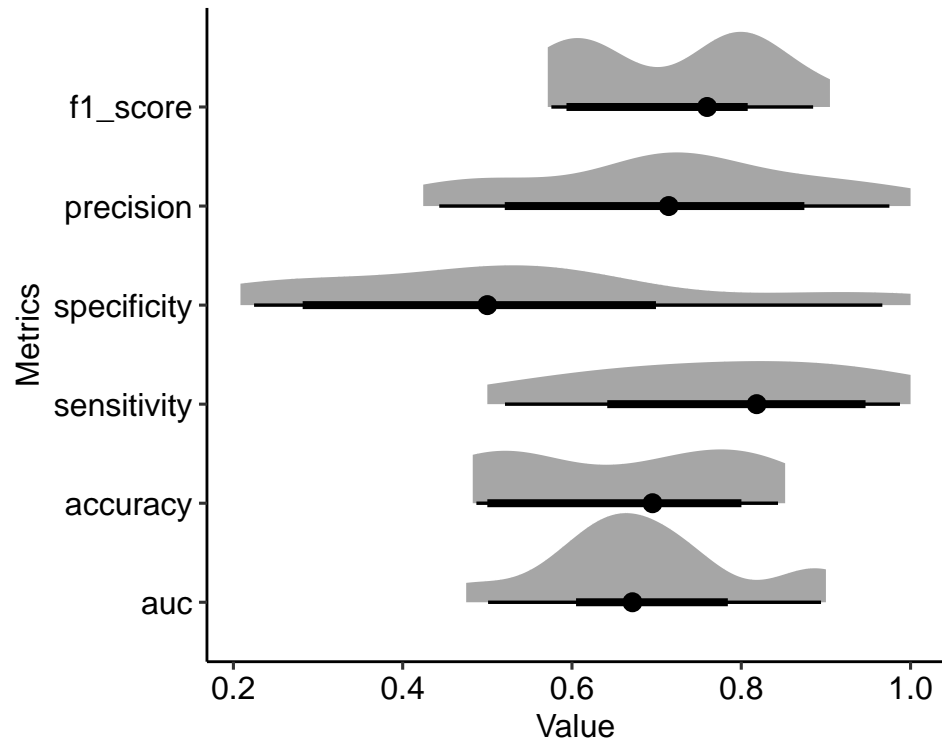

Supplement: Supplementary 1 — Tables S1 to 12 Figs. S1 to S5 [file csbj.0065.f1.zip › Figure_S3.pdf]

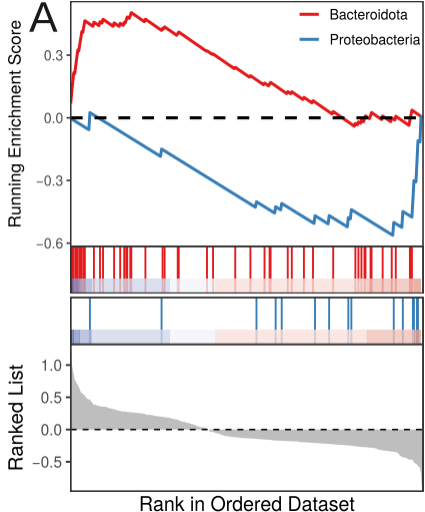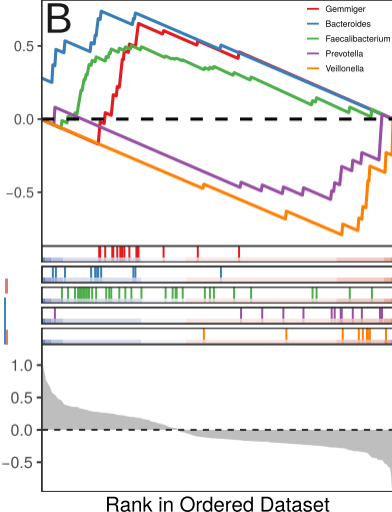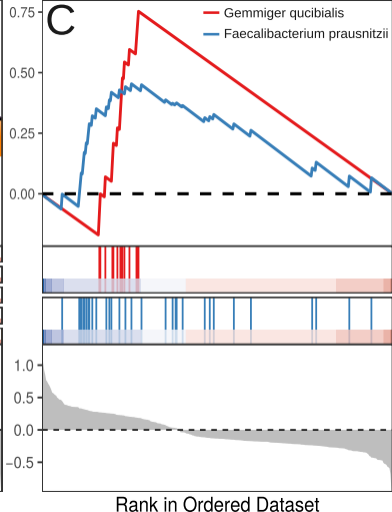

Supplement: Supplementary 1 — Tables S1 to 12 Figs. S1 to S5 [file csbj.0065.f1.zip › Figure_S5.pdf]
